# Inhibition of Bcl6b promotes gastric cancer by amplifying inflammation in mice

**DOI:** 10.1186/s12964-019-0387-6

**Published:** 2019-07-09

**Authors:** Wang-Yu Cai, Ling-Yun Lin, Lin Wang, Li Yang, Guo-Dong Ye, Qiang Zeng, Jia Cheng, Yuan-Yuan Xie, Mao-Li Chen, Qi-Cong Luo

**Affiliations:** 1 Department of Gastrointestinal Surgery, Zhongshan Hospital, Xiamen University, No. 201-209 Hubinnan Road, Xiamen, 361004 Fujian Province China; 2 Department of Oncology, Zhongshan Hospital, Xiamen University, No. 201-209 Hubinnan Road, Xiamen, 361004 Fujian Province China; 3 Laboratory of Xiamen Cancer Hospital, The First Affiliated Hospital of Xiamen University, No.55 Zhenhai Road, Xiamen, 361003 Fujian Province China; 4 Institute of Gastrointestinal Oncology, School of Medicine, Xiamen University, Xiamen, Fujian China; 5 Department of Pathology, School of Medicine, Jinan University, Guangzhou, Guangdong China; 6 Xiamen LifeInt Technology Co., Ltd., Xiamen, Fujian China

**Keywords:** Gastric cancer, Bcl6b, Mouse gastric cancer model, Inflammation, 5-Aza therapy

## Abstract

**Background:**

Chronic gastritis has been demonstrated to be a key cause of gastric cancer (GC), and control of gastric inflammation is regarded as an effective treatment for the clinical prevention of gastric carcinogenesis. However, there remains an unmet need to identify the dominant regulators of gastric oncogenesis-associated inflammation in vivo.

**Methods:**

The mouse model for the study of inflammation-associated GC was induced by Benzo[a]pyrene (BaP) intragastric administration in *Bcl6b*^−/−^ and wildtype mice on a C57BL/6 background. 5-Aza-2′-deoxycytidine (5-Aza), the demethylation drug, was intraperitoneally injected to restore Bcl6b expression. Human GC tissue array was used to analyse patient survival based on BCL6B and CD3 protein expression.

**Results:**

Bcl6b was gradually downregulated by its own promoter hypermethylation in parallel to an increasing inflammatory response during the progression of BaP-induced gastric carcinogenesis in mice. Moreover, knockout of Bcl6b dramatically worsened the severity of gastric cancer and aggravated the inflammatory response in the BaP-induced mice GC model. Re-activation of Bcl6b by 5-Aza impeded inflammatory amplification and BaP-induced GC development, prolonging survival time in wildtype mice, whereas no notable curative effect occurred in *Bcl6b*^−/−^ mice with 5-Aza treatment. Finally, significant negative correlations were detected between the mRNA levels of BCL6B and inflammatory cytokines in human GC tissues; patients harbouring BCL6B-negetive and severe-inflammation GC tumours were found to exhibit the shortest survival time.

**Conclusions:**

Epigenetic inactivation of Bcl6b promotes gastric cancer through amplification of the gastric inflammatory response in vivo and offers a new approach for GC treatment and regenerative medicine.

**Electronic supplementary material:**

The online version of this article (10.1186/s12964-019-0387-6) contains supplementary material, which is available to authorized users.

## Background

As the fifth-most commonly diagnosed cancer and the third leading cause of cancer-related death, gastric cancer (GC) remains an important cancer worldwide. In particular, Asia is responsible for the largest GC incidence and mortality [1]. Chronic gastritis, the persistent inflammation of the gastric mucosa, is a well-identified cause of GC [2]. Currently, the effective control of gastric mucosal inflammation has highlighted the importance of GC inhibition [3].

*H. pylori* infection and carcinogenic intake are key risk factors of inflammation associated gastric oncogenesis [4–6]. During chronic inflammation, the pronounced release of cytokines and chemokines, including interleukin-1 beta (IL-1β), tumour necrosis factor alpha (TNF-α), interleukin-8 (IL-8), and interleukin-6 (IL-6) [7, 8], drive the infiltration of neutrophils, macrophages and CD3^+^ T cell into the gastric mucosa [9]. Despite the well-known causal relationship between chronic gastritis and GC [10], the key regulators of gastric oncogenesis-associated inflammation have not been completely defined.

B-cell CLL/lymphoma 6 member B (*BCL6B*), also known as *BAZF*, is a homologue of the human proto-oncogene B-cell CLL/lymphoma 6 (*BCL6*) [11]. BCL6B has a 94% identical amino acid sequence at the zinc finger motifs and a 65% identical BTB/POZ domain to BCL6, thus, they bind to similar target DNA sequences to act as transcriptional repressors. However, the tissue expression pattern and pathological function of BCL6B differ from that of BCL6 [12–14]. In recent studies, BCL6B has been identified as a novel tumour suppressor, which is silenced or downregulated by promoter hypermethylation and is associated with poor survival in colorectal carcinoma [15], hepatocellular carcinoma [16, 17] and GC [18–20]. In terms of a molecular mechanism, several lines of evidence have revealed that BCL6B interacts with the Notch, STAT, p53 and PI3K/AKT signalling pathways, all of which may be involved in inflammatory response regulation in cancer cells [15, 16, 21, 22]. In vivo, Bcl6b inhibits hepatocellular inflammation to obstruct liver damage and fibrogenic induction in liver-specific *Bcl6b* transgenic rats [23]. However, whether BCL6B functions as a key tumour suppressor and inflammatory regulator during the progression of gastric oncogenesis in vivo requires further study.

In order to investigate the role of Bcl6b in gastric tumourigenesis, we assessed Bcl6b expression and the degree of inflammation during the progression of Benzo[a]pyrene (BaP)-induced gastric carcinogenesis in mice. Based on this established mouse GC model, we induced gastric tumours within wildtype and *Bcl6b*^−/−^ mice, and then treated with or without the demethylation drug 5-Aza-2′-deoxycytidine (5-Aza) to induce Bcl6b reactivation. We found that eradication of Bcl6b promoted gastric carcinogenesis by amplifying inflammation in mice and that BCL6B was associated with inflammation and survival in GC patients and mice. These results highlight the role of BCL6B in the modulation of malignant GC phenotypes in vivo and provide a potential target to develop a new therapeutic strategy for gastric cancer treatment.

## Methods

### Animals and treatments

*Bcl6b*^−/−^ mice on a C57BL/6 background were bred by Cyagen (Guangzhou, China). C57BL/6 mice were obtained from the Xiamen University Laboratory Animal Centre (Xiamen University, Xiamen, China). Mice had access to a standard chow diet and water ad libitum and were caged in a pathogen-free environment. All mice in our experiments were gender and age matched. BaP (Sigma-Aldrich, St Louis, MO, USA) was freshly prepared in sunflower oil (Sigma-Aldrich, St Louis, MO, USA) at a concentration of 5 mg/ml. At postnatal day 40–45 (P40–45), mice were treated with intragastric administration of 0.01 ml/g body weight BaP two times weekly for five weeks. The first intragastric dose was at week 0. Mice were randomized to receive either an intraperitoneal injection of 5-Aza (0.5 mg/kg body weight, twice a week; Sigma-Aldrich, St Louis, MO, USA) or saline during week 10–25 for 15 weeks. Mice were euthanized, and stomach tissues were collected for examination at specific times. All mice were used in accordance with the guidelines of the Institutional Animal Care and Use Committee of the Xiamen University Laboratory Animal Centre.

### RNA extraction and quantitative PCR analysis

Total RNA from mouse tissues was extracted using TRIzol (cat. no.: 15596026, Invitrogen, Thermo Fisher Scientific, MA, USA) and was quantified via NanoDrop (Thermo Fisher Scientific, MA, USA). Equal amounts of RNA were reverse-transcribed with the HiFi-MMLV cDNA Kit from CWBIO (cat. no.: CW0744, CoWin Biotech, Beijing, China) to produce cDNA according to the manufacturer’s protocol. Quantitative PCR (qPCR) was performed using a CFX96 Touch Real-Time PCR Detection System (Bio-Rad, CA, USA) with the UltraSYBR Mixture from CWBIO (cat. no.: CW0956, Beijing CoWin Biotech, Beijing, China) according to the manufacturer’s protocol. The expression levels were quantified using the 2^-ΔΔCt^ (where Ct is the threshold cycle) method. Eukaryotic 18S rRNA endogenous control was used as an internal standard. The following primer sequences for quantitative PCR were used:

18S: 5′- GTCTGTGATGCCCTTAGATG − 3′ (forward),

5′- AGCTTATGACCCGCACTTAC − 3′ (reverse);

hTNF-α: 5′- AGCCTGTAGCCCATGTTGTAGC − 3′ (forward),

5′- CCTTGGCCCTTGAAGAGGAC − 3′ (reverse);

hIL-8: 5′- TTGGCAGCCTTCCTGATTTCT − 3′ (forward),

5′- GGTCCACTCTCAATCACTCTCA − 3′ (reverse);

hIL-1β: 5′- GAGCTCGCCAGTGAAATGATG − 3′ (forward),

5′- TCGGAGATTCGTAGCTGGATG − 3′ (reverse);

hBCL6B: 5′- CTACGTCCGCGAGTTCACTC − 3′ (forward),

5′- CCCGGAAAATTGAATAGAAG − 3′ (reverse);

mTNF-α: 5′- TGACAAGCCTGTAGCCCACG − 3′ (forward),

5′- GGCAGCCTTGTCCCTTGAA − 3′ (reverse);

mIL-8: 5′- CAAGGCTGGTCCATGCTCC − 3′ (forward),

5′- TGCTATCACTTCCTTTCTGTTGC − 3′ (reverse);

mIL-1β: 5′- GAAATGCCACCTTTTGACAGTG − 3′ (forward),

5′- TGTTGATGTGCTGCTGCGAG − 3′ (reverse);

mBcl6b: 5′- TCCTCCGACGTGCTTAGCAAT − 3′ (forward),

5′- GGCCCCGGAAAATTGAATAGA − 3′ (reverse).

### Enzyme-linked immunosorbent assay

Mouse serum levels of TNF-α and IL-1β were assessed and quantified using enzyme-linked immunosorbent assay (ELISA) kits (TNF-α: cat. no.: MTA00B, R&D systems, Minneapolis, USA; IL-1β: cat. no.: 88–7013-22, Thermo Fisher Scientific, MA, USA) according to the manufacturers’ protocol. Enzyme concentration was quantified by measuring the optical density at a wavelength of 450 nm (OD450) using a spectrophotometer (Thermo Fisher Scientific, MA, USA).

### Western blotting

Mouse tissues were lysed with SDS lysis buffer. Anti-mBcl6b (cat. no.: ab87228, Abcam, Cambridge, MA, USA) and anti-β-actin (cat. no.: A1978, Sigma-Aldrich, Darmstadt, Germany) were used for immunoblotting. β-Actin was used as an internal control.

### Morphological observation

The stomach of the mice was cut off from the greater curvature and placed on a stereomicroscope. Turn on the bottom light source, count and measure tumour nodules larger than 0.5 mm in diameter according to the lens scale. The average number of tumour nodules per tumour bearing mouse was calculated as tumour number. The average maximal diameter of tumour nodule per tumour bearing mouse was calculated as max size. Tumour incidence was the number of mice carrying at least 1 tumour nodule expressed as percentage incidence. Meanwhile, H&E staining and Pan-Cytokeratin immunostaining in the gastric tissue of mice were further used for qualitative analysis of gastric tumours.

### H&E staining

For histologic analysis, mouse tissues were fixed with 10% neutral buffered formalin overnight, embedded in paraffin, and cut into 4.5 μm sections using a microtome (Leica RM2235; Leica Microsystems). After dewaxing and rehydration, the sections were stained in haematoxylin solution (cat. no.: D006, Nanjing Jiancheng Bioengineering Institute, Nanjing, China) for 3 min, washed in running tap water and then differentiated in 1% acid alcohol for 30 s, followed by counterstaining with eosin (cat. no.: D006, Nanjing Jiancheng Bioengineering Institute, Nanjing, China) for 30 s. Gastric tumours were identified and quantified on sections.

### Immunohistochemistry

All tissues were fixed in 10% neutral buffered formalin, paraffin embedded, and sectioned. After dewaxing and rehydration, antigen retrieval was performed by boiling in citrate buffer (pH 6.0) for 20 min. Immunostaining was performed using the Maxim UltraSensitive SAP Kit (cat. no.: Kit9710, Maxim Biological Technology, Fuzhou, China). Sections were pre-treated with peroxidase blocking buffer for 10 min at room temperature. After treatment with blocking buffer (5% normal goat serum in PBS) for 10 min at room temperature, sections were incubated with anti- hBCL6B (1:500 dilution; cat. no.: ab180084, Abcam, Cambridge, MA, USA), anti- mBcl6b (1:500 dilution; cat. no.: ab87228, Abcam, Cambridge, MA, USA), anti-CD3 (1:300 dilution; cat. no.: sc-20047, Santa Cruz Biotechnology, Texas, USA), anti-Ki-67 (cat. no.: 790–4276, Ventana Medical systems, AZ, USA), or anti- Pan-Cytokeratin (cat. no.: IHC-MO67, Guangzhou ambip pharmaceutical technology, Guangzhou, China) in blocking buffer. Sections were then incubated with a secondary antibody followed by 3,3′-diaminobenzidine (DAB) staining with a DAB kit (cat. no.: DAB2031, Maxim Biological Technology, Fuzhou, China). Images were obtained using a computerized imaging system (Leica Microsystems, Imaging Solutions Ltd., Cambridge, UK). Identical settings were used for each photograph using Leica QWin Plus v3 software. To strengthen our main conclusion and further investigate the clinical significance of BCL6B and CD3 in GC, we performed tissue microarray analysis and classified all tumours that presented with more than 5% of cells over the threshold as BCL6B or CD3 positive.

### Bisulphite sequencing PCR

DNA purified from mouse tissues was subjected to bisulphite sequencing PCR using a DNA Methylation Kit (cat. no.: CW2140, Beijing CWbio Biotech, Beijing, China) following the manufacturer’s instructions. PCR primers were designed targeting the CpG sites of the mouse *Bcl6b* gene (starting 80-bp upstream of the mouse *Bcl6b* transcription start site (TSS) and ending 100-bp downstream of the TSS):

5′- GGGTTTATTATTTGGAGAGT − 3′ (forward), 5′- TTAATTCTTACCCCTATCCC − 3′ (reverse).

### Human gastric cancer samples

Tissue microarrays from 186 GC patients (HStmA180Su09/HStmA180Su13) and cDNA microarrays from 45 GC patients (cDNA-HStmA030CS01/ cDNA-HStmA060CS01) were purchased from Shanghai Outdo Biotech Co., Ltd. (Shanghai, China). The studies were conducted in accordance with the International Ethical Guidelines for Biomedical Research Involving Human Subjects (CIOMS), and the research protocols were approved by the Clinical Research Ethics Committee of Zhongshan Hospital of Xiamen University.

### Statistical analysis

Statistical analyses were performed with GraphPad Prism 6.0. Data are presented as the mean ± SD. The significance of the mean values between two groups was analysed by Student’s *t* test. Pearson correlation analysis was performed to determine the correlation between two variables. Survival analysis was performed using a log-rank test. *P* < 0.05 was considered statistically significant.

## Results

### The expression patterns of Bcl6b and inflammatory factors correlated with the progression of BaP-induced gastric carcinogenesis

**Fig. 1:**
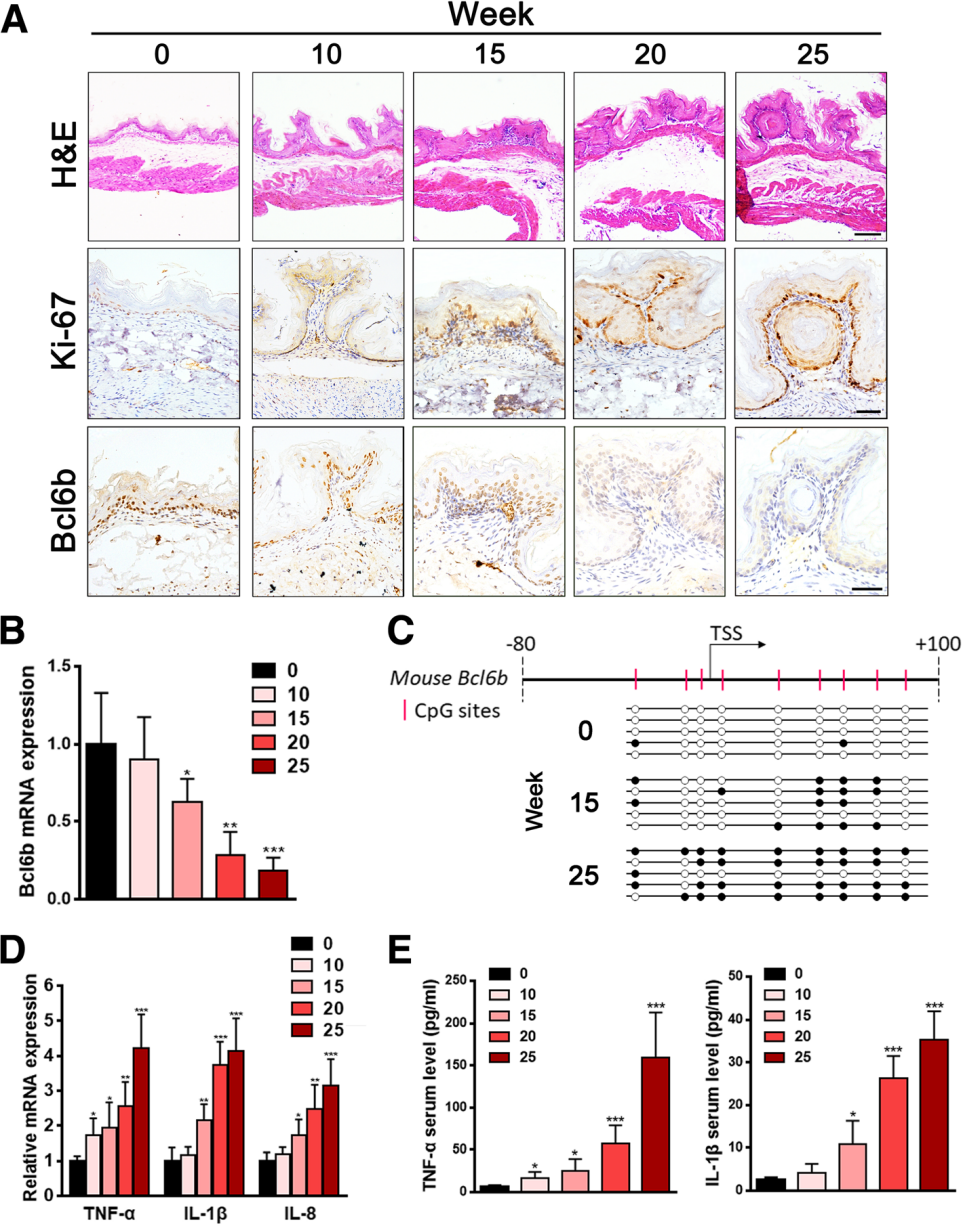
Bcl6b was downregulated by promoter methylation along with activated inflammation throughout the progression of BaP-induced gastric carcinogenesis in mice. (**a**) Representative images showing H&E staining of stomachs (Scale bar, 100 μm) and immunostaining of Ki-67 and Bcl6b expression in mouse gastric samples (Scale bar, 50 μm) throughout the progression of BaP-induced carcinogenesis. (**b**) qRT-PCR revealed Bcl6b mRNA levels in mouse gastric samples during the progression of BaP-induced carcinogenesis. (**c**) A typical CpG island spans the promoter region of mouse *Bcl6b*. Each vertical bar represents a single CpG site. The transcription start site (TSS) is indicated by a curved arrow. Bisulphite genomic sequencing (BGS) analysis revealed the methylation status of *Bcl6b* in mouse gastric samples during the progression of BaP-induced carcinogenesis. Filled circles: methylated CpG sites; open circles: unmethylated CpG sites. (**d**, **e**) qRT-PCR revealed the TNF-α, IL-1β and IL-8 mRNA levels in mouse gastric samples (D) and ELISA revealed the serum protein levels of TNF-α and IL-1β (**e**) during the progression of BaP-induced gastric cancer in mice. Data are presented as the mean ± SD; *n* = 5 per group. *P < 0.05; **P < 0.01; ***P < 0.001; unpaired two-tailed Student’s *t* tests

To elucidate whether Bcl6b is regulated in BaP-induced gastric carcinogenesis in vivo*,* a mouse gastric cancer model was established in P40–45 C57BL/6 mice by intragastric administration of BaP (0.05 mg/g) twice weekly for five weeks. Throughout the progression of BaP-induced gastric carcinogenesis, gradual pathologic alterations and eventual tumour granules were monitored by H&E staining of mouse stomach tissues (Fig. 1a). Simultaneously, the expression of Ki-67, a general proliferation marker that can be used to characterize gastric tumourigenesis, was dramatically elevated throughout this progression (Fig. 1a). Bcl6b protein expression in mouse gastric mucosa was detected by immunostaining and gradually decreased during gastric carcinogenesis (Fig. 1a). Furthermore, quantitative PCR (qPCR) revealed a similar trend towards downregulated expression of Bcl6b messenger RNA (mRNA) throughout this process (Fig. 1b). Since *BCL6B* has been reported to be silenced via its promoter hypermethylation in human gastric cancer [19], bisulphite genomic sequencing (BGS) analysis was performed, which subsequently revealed that the methylation status of the *Bcl6b* promoter was elevated during BaP-induced gastric carcinogenesis (Fig. 1c). We next investigated whether BaP-induced gastric carcinogenesis was accompanied by an elevated inflammatory response. Quantitative analysis of the mRNA levels of TNF-α, IL-1β and IL-8 in gastric mucosal tissues showed that the inflammatory response was significantly activated in parallel with tumourigenesis (Fig. 1d). TNF-α and IL-8 protein levels in mouse serum as detected by ELISA also displayed a gradually increasing trend during BaP induction (Fig. 1e). Taken together, the above results suggest that Bcl6b was downregulated by promoter methylation along with activated inflammation in BaP-induced mouse gastric cancer model.

### Eradication of Bcl6b aggravates gastric tumourigenesis and amplifies inflammation in BaP-treated mice

**Fig. 2:**
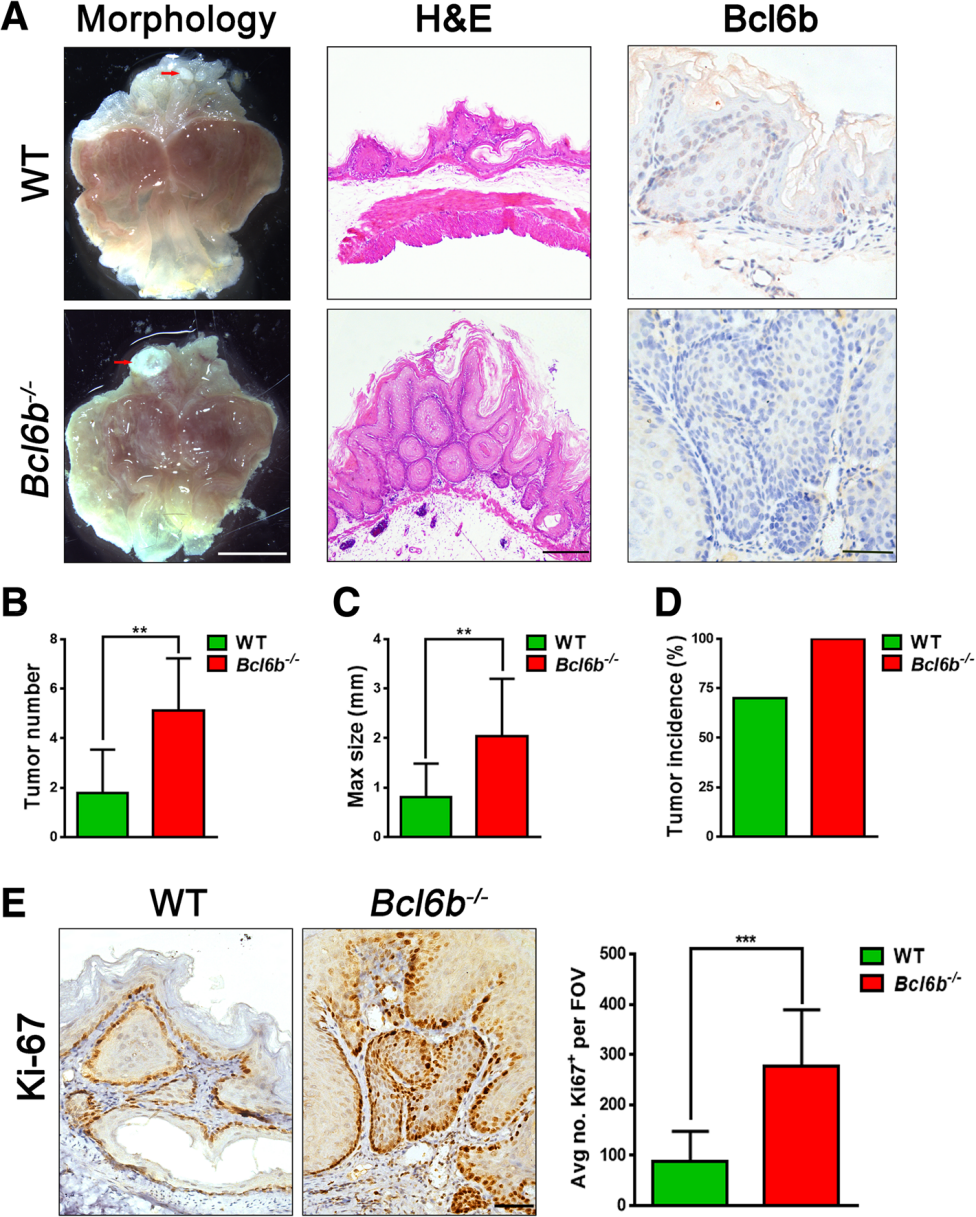
*Bcl6b*^−/−^ mice developed more gastric tumours following BaP treatment. (**a**) Representative images showing gross morphology (Scale bar, 5 mm), H&E staining (Scale bar, 100 μm) and immunostaining of Bcl6b expression (Scale bar, 50 μm) from stomachs harbouring BaP-induced tumours (27 week) from *Bcl6b*^−/−^ mice and WT controls. The gastric tumour granules were indicated by red arrows. (**b**-**d**) Tumour number (**b**), maximal size (**c**), and tumour incidence (**d**) in the stomachs of WT and *Bcl6b*^−/−^ mice after intragastric administration of BaP (27 week). (**e**) Representative images and quantification of Ki-67-immunopositive cells in Bap-induced gastric tumours (27 week) from *Bcl6b*^−/−^ mice and WT controls. Scale bar, 50 μm. Data are presented as the mean ± SD; *n* = 10 per group. **P < 0.01; ***P < 0.001; unpaired two-tailed Student’s *t* tests

To elucidate whether Bcl6b has any effect on inflammation associated gastric tumourigenesis in vivo, BaP-induced mouse gastric cancer models were established in *Bcl6b*^−/−^ and wildtype mice. We then sacrificed the mice and identified and quantified the gastric tumour load at 27 weeks after the initial intragastric BaP administration. Bcl6b expression in the gastric mucosa as detected by immunostaining were eradicated thoroughly within *Bcl6b*^−/−^ mice, and trace Bcl6b was expressed in wildtype mice upon BaP treatment (Fig. 2a, right). Images of gross gastric morphology (Fig. 2a, left), H&E staining (Fig. 2A, middle) and Pan-Cytokeratin immunostaining (Additional file 1: Figure S1) showed that tumour number (Fig. 2b) and maximum size (Fig. 2c) were significantly higher in Bap-treated *Bcl6b*^−/−^ mice versus the control group. A greater than 30% increase in the incidence of tumour development upon BaP treatment was also observed in *Bcl6b*^−/−^ mice (Fig. 2d). Moreover, we evaluated the number of Ki-67 immunopositive cells in the gastric mucosa of BaP-treated mice; *Bcl6b*^−/−^ mice exhibited a dramatic 2-fold increase in Ki-67 labelling relative to control mice (Fig. 2e). Thus, these results indicate that eradication of Bcl6b aggravates BaP-induced gastric tumourigenesis in vivo.

**Fig. 3:**
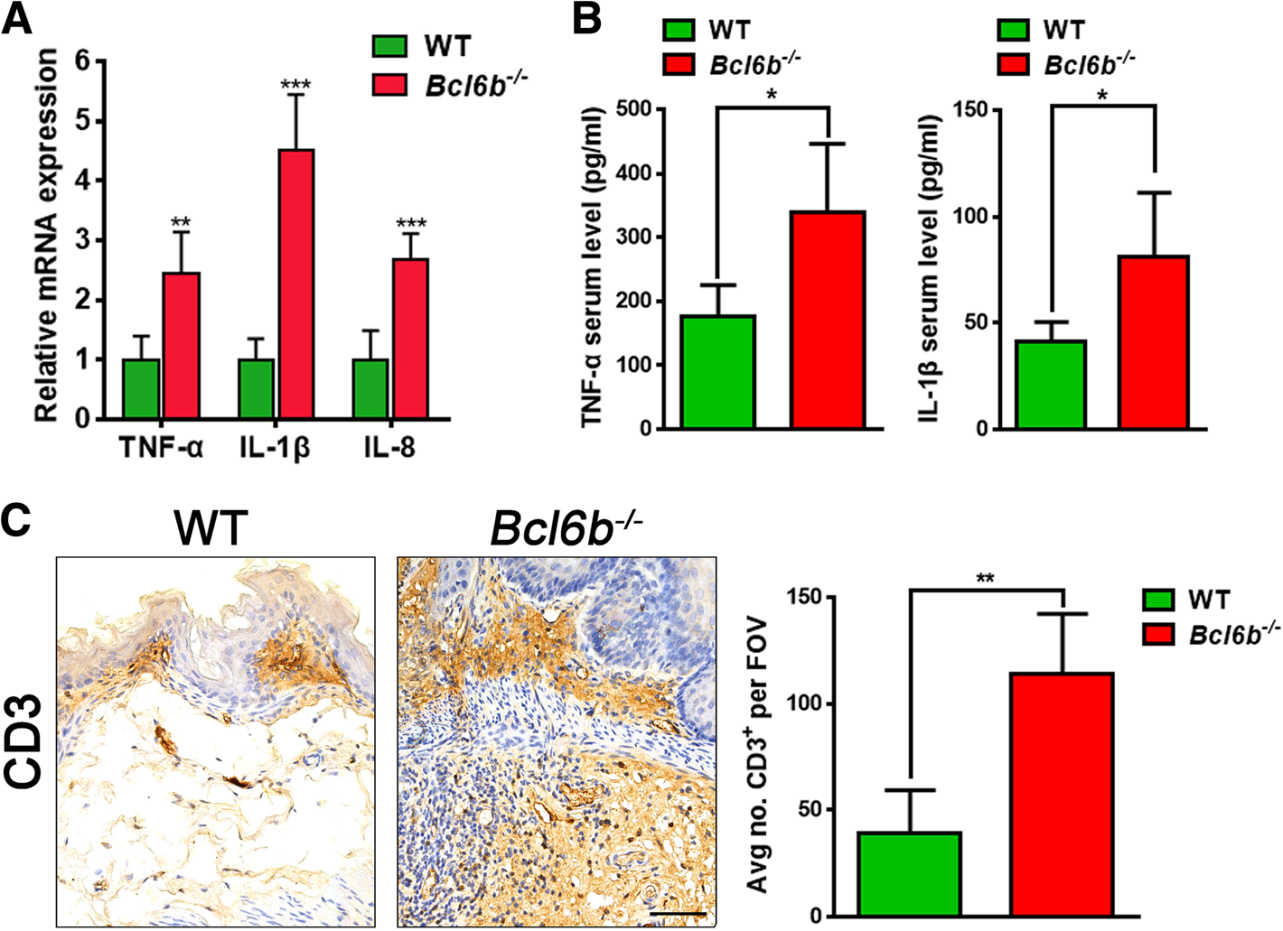
Ablation of *Bcl6b* amplified inflammation upon BaP treatment. (**a**) qRT-PCR revealed TNF-α, IL-1β and IL-8 mRNA levels in Bap-induced gastric tumours (27 week) from *Bcl6b*^−/−^ mice and WT controls. (**b**) ELISA revealed serum protein levels of TNF-α and IL-1β in WT and *Bcl6b*^−/−^ mice after intragastric administration of BaP (27 week). (**c**) Representative images and quantification of CD3-immunopositive cells in Bap-induced gastric tumours (27 week) from *Bcl6b*^−/−^ mice and WT controls. Scale bar, 50 μm. Data are presented as the mean ± SD; n = 5 per group. *P < 0.05; **P < 0.01; ***P < 0.001; unpaired two-tailed Student’s *t* tests

Given that upregulated gastric inflammatory cytokines, such as TNF-α, IL-1β and IL-8 increase the risk of GC [7, 24], we next investigated whether GC-sensitive *Bcl6b*^−/−^ mice had increased levels of inflammatory factors upon BaP treatment. Compared to control mice, the mRNA levels of inflammatory cytokines TNF-α, IL-1β and IL-8 in the gastric mucosa were dramatically elevated in BaP-treated *Bcl6b*^−/−^ mice (Fig. 3a). Additionally, ELISA revealed that TNF-α and IL-1β serum protein levels were also significantly increased in BaP-treated *Bcl6b*^−/−^ mice (Fig. 3b). Since inflammatory cytokines drive CD3-positive inflammatory T-cell infiltration into the gastric mucosa during gastric carcinogenesis, we next evaluated the number of CD3 immunopositive cells in the gastric mucosa of BaP-treated mice. As expected, BaP-treated *Bcl6b*^−/−^ mice exhibited a dramatic 2-fold increase in CD3 labelling relative to control mice (Fig. 3c). Taken together, these data suggest that the inflammatory response is amplified by Bcl6b ablation, which may aggravate BaP-induced gastric carcinogenesis in vivo.

### 5-Aza re-activates Bcl6b and attenuates the inflammatory response in BaP-treated mice

**Fig. 4:**
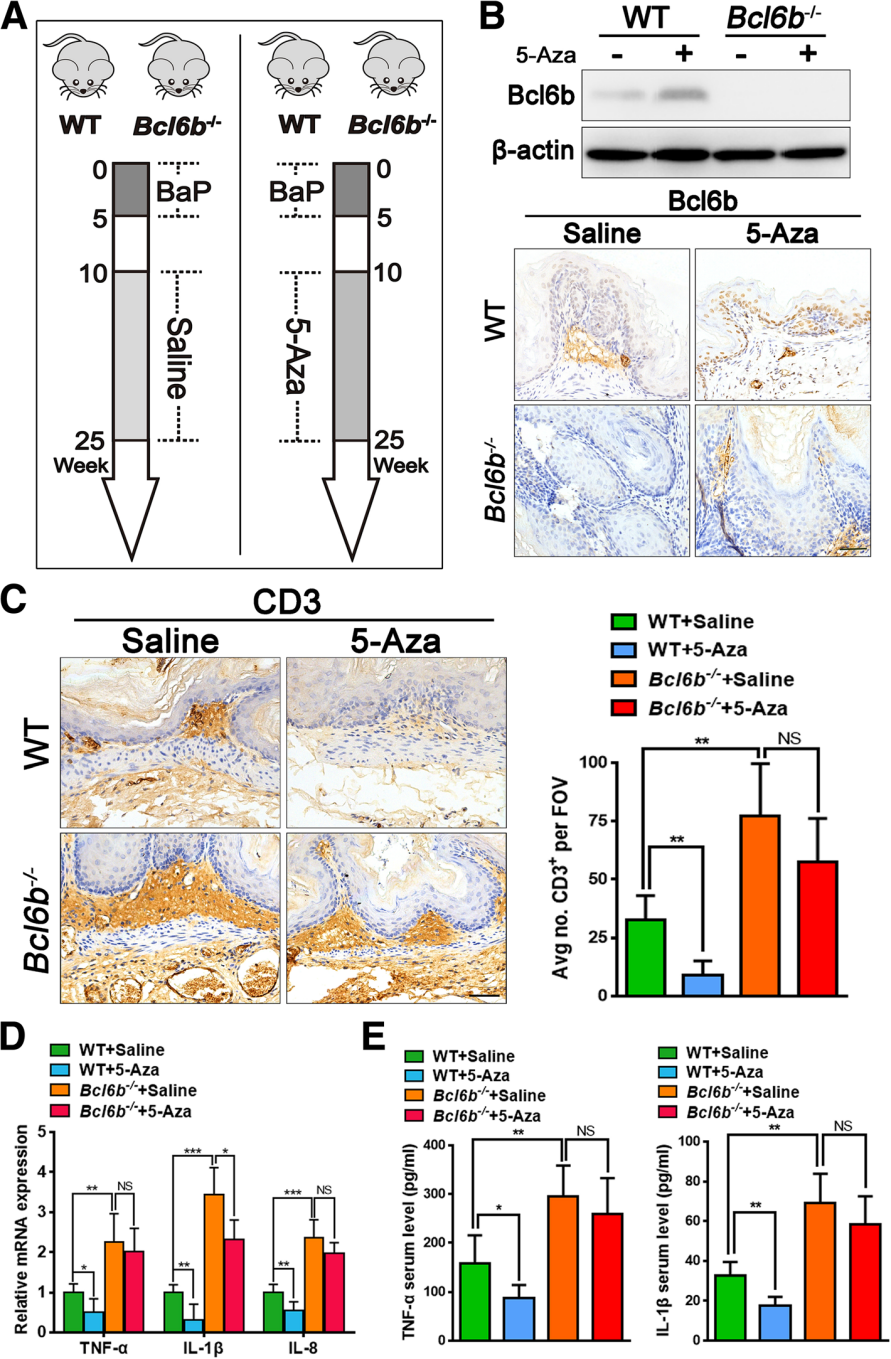
5-Aza re-activated Bcl6b and inhibited inflammatory responses. (**a**) WT and *Bcl6b*^−/−^ mice were given Bap for gastric cancer induction during weeks 10–25 of treatment with or without the demethylation drug 5-Aza at a dose of 0.5 mg/kg for 15 consecutive weeks. (**b**) Representative images showing immunostaining and Western blotting of Bcl6b expression in Bap-induced gastric tumours (25 week) from *Bcl6b*^−/−^ mice and WT controls with or without 5-Aza treatment. Scale bar, 50 μm. (**c**) Representative images and quantification of CD3-immunopositive cells in Bap-induced gastric tumours (25 week) from *Bcl6b*^−/−^ mice and WT controls with or without 5-Aza treatment. Scale bar, 50 μm. (**d**) qRT-PCR revealed TNF-α, IL-1β and IL-8 mRNA levels from stomachs harbouring BaP-induced tumours (25 week) from *Bcl6b*^−/−^ mice and WT controls with or without 5-Aza treatment. (**e**) ELISA revealed serum protein levels of TNF-α and IL-1β in WT and *Bcl6b*^−/−^ mice after intragastric administration of BaP (25 week) with or without 5-Aza treatment. Data are presented as the mean ± SD; n = 5 per group. *P < 0.05; **P < 0.01; ***P < 0.001; NS, not significant; unpaired two-tailed Student’s *t* tests

As Bcl6b was dramatically downregulated by promoter methylation in our BaP-induced mouse gastric cancer model, we therefore hypothesized that the demethylation drug 5-Aza may ameliorate the BaP-induced inflammatory response during gastric carcinogenesis through re-activated Bcl6b. To test this hypothesis, P40–45 C57BL/6 *Bcl6b*^−/−^ and wildtype mice were given BaP to induce gastric cancer as before during week 10–25 with or without the demethylation drug 5-Aza at a dose of 0.5 mg/kg for 15 consecutive weeks (Fig. 4a). Mice were euthanized, and stomach tissues were collected for examination at the end of 5-Aza treatment at week 25. As expected, Western blot and immunostaining results showed that Bcl6b protein expression was remarkably re-activated upon 5-Aza treatment in the gastric mucosa of the wildtype group, while 5-Aza treatment had no effect on Bcl6b activation in the *Bcl6b*^−/−^ mice (Fig. 4b). Next, gastric mucosal Inflammatory cytokines such as TNF-α, IL-1β and IL-8 were evaluated by qPCR and showed significantly lower expression in 5-Aza-treated wildtype mice compared to saline controls, while 5-Aza had only a minor effect in *Bcl6b*^−/−^ mice (Fig. 4d). Similar results were achieved when we performed ELISA to detect serum protein levels of TNF-α and IL-1β (Fig. 4e). In addition, CD3^+^ inflammatory T cells were found to be reduced by 75% in the above Bcl6b-re-activated wildtype group compared to saline controls, whereas no significant change in the *Bcl6b*^−/−^ mice was observed (Fig. 4c). Therefore, these results suggest that 5-Aza can inhibit BaP-induced gastric inflammation dependent on Bcl6b re-activation in vivo*.*

### 5-Aza ameliorates BaP-induced gastric oncogenesis depended on Bcl6b in vivo

**Fig. 5:**
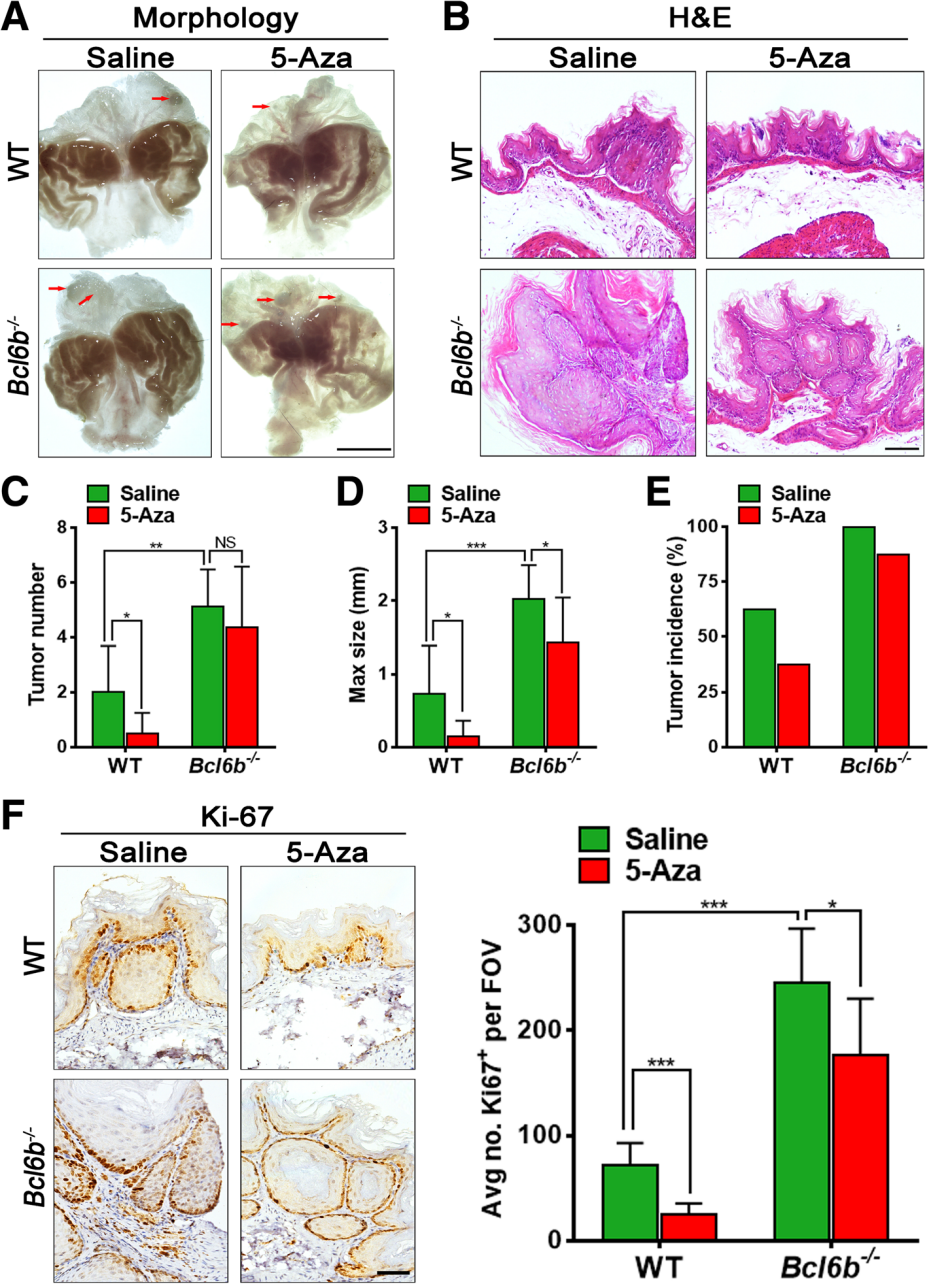
5-Aza prevented BaP-induced gastric tumour development in a Bcl6b–dependent manner. (**a**, **b**) Representative images showing gross morphology (Scale bar, 5 mm) (**a**) and H&E staining (Scale bar, 100 μm) (**b**) of stomachs harbouring BaP-induced tumours (25 week) from *Bcl6b*^−/−^ mice and WT controls with or without 5-Aza treatment. The gastric tumour granules were indicated by red arrows. (**c**-**e**) Tumour number (**c**), maximal size (**d**), and tumour incidence (**e**) in stomachs from WT and *Bcl6b*^−/−^ mice after intragastric administration of BaP (25 week) with or without 5-Aza treatment. (**f**) Representative images and quantification of Ki-67-immunopositive cells in Bap-induced gastric tumours (25 week) from *Bcl6b*^−/−^ mice and WT controls with or without 5-Aza treatment. Scale bar, 50 μm. Data are presented as the mean ± SD; *n* = 8 per group. *P < 0.05; **P < 0.01; ***P < 0.001; NS, not significant; unpaired two-tailed Student’s *t* tests

**Fig. 6:**
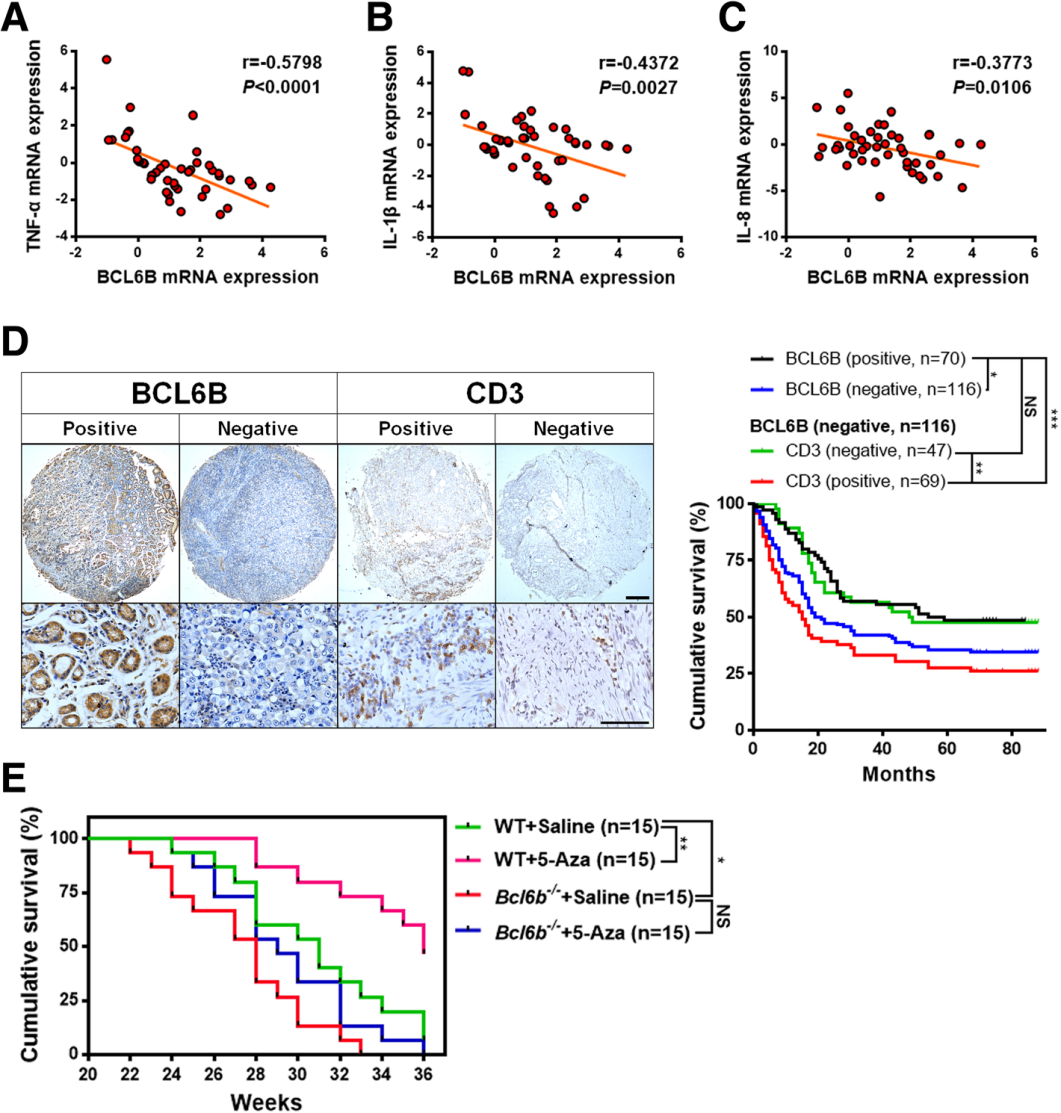
BCL6B was associated with inflammation and survival in GC patients and mice. (**a**-**c**) Negative correlations between BCL6B and TNF-α (**a**), IL-1β (B) and IL-8 (**c**) mRNA levels in human gastric cancer tissues were measured using Pearson’s correlation test (r and *P* values are shown in the graphs, *n* = 45). (**d**) Representative immunostaining of BCL6B and CD3 in human gastric cancer tissue microarrays. Scale bar, 100 μm (top) and 20 μm (bottom). Kaplan-Meier survival curves from overall gastric cancer patients based on BCL6B protein expression and BCL6B-negative gastric cancer patients based on CD3 protein expression. Survival analysis was performed using a log-rank test. (**e**) Kaplan-Meier survival curves of WT and *Bcl6b*^−/−^ mice after intragastric administration of BaP to induce gastric cancer with or without 5-Aza treatment. Survival analysis was performed using a log-rank test. *P < 0.05; **P < 0.01; ***P < 0.001; NS, not significant

To further address whether 5-Aza has any beneficial effects on GC formation, we identified and quantified the gastric tumour load in BaP-treated *Bcl6b*^−/−^ mice and wildtype controls with or without 5-Aza treatment. As indicated by morphologic images (Fig. 5a), H&E staining (Fig. 5b) and Pan-Cytokeratin immunostaining (Additional file 2: Figure S2), the demethylation drug 5-Aza obviously decreased tumour number (Fig. 5c) and maximum tumour size (Fig. 5d) by greater than 70% in BaP-treated wildtype mice compared to saline controls, but only had a minor role in BaP-treated *Bcl6b*^−/−^ mice. A 25% reduction in the tumour incidence following 5-Aza treatment was observed in the wildtype mouse GC model compared to saline controls, while a minor curative effect (12.5% reduction) following 5-Aza treatment occurred in BaP-treated *Bcl6b*^−/−^ mice (Fig. 5e). Moreover, Ki-67 immunostaining showed that compared to the 28% reduction in Ki-67 labelling following 5-Aza treatment in BaP-treated *Bcl6b*^−/−^ mice, Ki-67 positive cells were decreased by 65% upon 5-Aza treatment in BaP-treated wildtype mice relative to saline controls (Fig. 5f). Finally, Kaplan-Meier survival curves showed that survival time was dramatically shorter in *Bcl6b*^−/−^ mice upon BaP treatment compared to the wildtype mice (Fig. 6e). Strikingly, 5-Aza treatment significantly lengthened the survival time of BaP-treated wildtype mice, however, no significant prolongation of survival time following 5-Aza treatment was observed in the BaP-treated *Bcl6b*^−/−^ group (Fig. 6e). Taken together, these results demonstrated that 5-Aza effectively prevented BaP-induced gastric tumour development in a Bcl6b-dependent manner.

### BCL6B downregulation combined with a severe inflammatory response correlates with poor survival in GC patients

To strengthen our main conclusion and further clarify the clinical significance of BCL6B and inflammation in GC, cDNA microarrays from 45 GC patients were first used to investigate the mRNA expression pattern of BCL6B and inflammatory cytokines. Notably, correlation analyses revealed strong inverse correlations between the overall expression levels of BCL6B and the inflammatory cytokines TNFα (Fig. 6a), IL-1β (Fig. 6b), and IL-8 (Fig. 6c) in the 45 GC patient cohort. Next, we performed immunohistochemical staining of BCL6B and CD3 on GC tissue microarrays containing 186 GC specimens that had long-term clinical follow-up records. As the typical images show in Fig. 6d, all tumours presenting with greater than 5% positive staining of cells over the threshold were defined as BCL6B or CD3 positive. Subsequent Kaplan-Meier survival analysis showed that the GC tissue expression levels of BCL6B protein were inversely correlated with the 5-year survival rate of GC patients. BCL6B-negative GC patients had a median survival time of 56.5 months compared to BCL6B-positive GC patients who had a median survival time of 20.0 months (Fig. 6d), which is consistent with previous reports [18, 19] and our own survival results in mice as shown in Fig. 6e. Remarkably, IHC analysis showed that among BCL6B-negative GC patients, those with CD3-positive expression exhibited the poorest survival (median survival time = 15.0 months), whereas BCL6B-negative GC patients with CD3-negative expression showed marginally different survival with a median survival time of 48.5 months (Fig. 6d). Taken together, the above results provide evidence that BCL6B is significantly negatively related to the degree of inflammation in GC and that negative BCL6B expression and a severe inflammatory response contribute jointly to the poor survival of GC patients.

## Discussion

In this study, we used *Bcl6b*-deficient mice and 5-Aza-treated mice to evaluate the role of Bcl6b in BaP induced gastric inflammation and GC development. This study provided compelling evidence that Bcl6b functions as a tumour suppressor to inhibit GC and suggested a novel inflammatory strategy to control GC. Previous studies have showed that inflammation is a hallmark of cancer and a pro-tumourigenic factor [25, 26]. Furthermore, gastritis stage is used to quantify GC risk [27] and GC patients with a severe inflammatory response exhibit poor survival [28]. Thus, effective inflammation control has been recognized as both a preventative and treatment strategy for GC [29–31]. In the present study, we found that Bcl6b was gradually downregulated by its own promoter hypermethylation in parallel to an increased inflammatory response during the progression of BaP-induced gastric carcinogenesis in mice. Moreover, knockout of *Bcl6b* amplified the inflammatory response and aggravated gastric tumourigenesis in mice upon BaP treatment. Re-activation of Bcl6b by the demethylation drug 5-Aza could impede inflammatory amplification and Bap-induced GC development, further prolonging the survival time in wildtype mice, whereas no notable curative effect was observed in *Bcl6b*^−/−^ mice with 5-Aza treatment. Analyses of clinical GC specimens showed that negative BCL6B expression together with a severe inflammatory response resulted in the poorest survival in GC patients. We therefore conclude that Bcl6b plays a crucial role in regulating inflammation-associated GC initiation and development in vivo and that 5-Aza can be used as an anti-tumour compound through re-activation of Bcl6b.

Given that the inflammatory response has been reported to modulate GC development [32–34] and that BaP is a carcinogen that is most likely ingested during daily human life [35, 36], BaP can induce cancerous lesions in the human stomach and result in gastric carcinogenesis by promoting a pro-inflammatory phenotype [5, 37–39]. In addition, feeding of mice with BaP has been an extensively applied method for the establishment of mouse GC models [40, 41]. Thus, BaP-induced mouse GC is therefore an ideal model for the study of inflammation-associated GC. Consistent with our observation in our mouse GC model induced by BaP gavage, inflammation gradually increased during gastric carcinogenesis with a corresponding decreasing trend for Bcl6b. In human GC samples, Bcl6b expression exhibited a negative correlation with inflammatory cytokines. Based on the previous report that BCL6B is an independent predictor of poor outcome in patients with GC [19]. Our study further indicates that combination of BCL6B expression and inflammatory response may improve the prognosis accuracy in GC patient survival. Moreover, our results showed that Bcl6b inhibition remarkably aggravated gastric inflammation in BaP-induced mice. These findings indicate that BCL6B functions as a key inflammatory regulator in the progression of gastric oncogenesis in vivo. However, the precise regulatory mechanisms underlying the relationship between BCL6B and inflammatory cytokines has not yet been elucidated. Several previous studies revealed that BCL6B interacts with the Notch, STAT, p53 and PI3K/AKT signalling pathways [15, 16, 21, 22], which may be involved in the regulation of the inflammatory response in cancer cells. Future studies should verify which core signalling pathway BCL6B depends on for the regulation of the inflammatory response in inflammation-associated GC development.

From a clinical point of view, both BCL6B and gastric inflammation should be precisely regulated within a suitable range to control normal development and homeostasis. However, this balance may be disrupted during carcinogenesis. It has been reported that BCL6B is silenced via its own promoter hypermethylation in GC [19], which is consistent with our observation that the methylated levels of the *Bcl6b* promoter were gradually up-regulated during the process of gastric tumourigenesis by BaP induction. In our study, which aimed to investigate an existing clinical drug to target Bcl6b in inflammation-associated GC in vivo. We used the DNA demethylation drug 2′-deoxy-5-azacytidine (5-Aza), which has been applied for the clinical treatment of multiple haematologic malignancies and solid tumours [42, 43]. Our results revealed that 5-Aza treatment effectively restored Bcl6b expression and dramatically blocked gastric inflammation and GC development. In contrast, 5-Aza treatment had a weak therapeutic effect on BaP-induced GC in *Bcl6b*^−/−^ mice. This study, along with another complementary reports [44, 45] confirmed that a specific panel of candidate genes (*BCL6B, GDF1, FBP1, BNIP3, CDX1, CHFR, MGMT, MLH1*, etc.) are aberrantly activated or silenced by methylation in stomach tumours. Our results in *Bcl6b*^−/−^ mice suggest that the therapeutic effect of 5-Aza on BaP-induced GC depends on the activation of Bcl6b, which highlights the key role of *BCL6B* methylation in the occurrence and development of GC. In clinical trials, the demethylation drug 5-Aza, which broadly targets DNA, showed high toxicity [46–48]. Thus, a novel anti-GC drug that can selectively target BCL6B with low toxicity is urgently needed.

## Conclusions

In summary, our study showed that BCL6B acts as a dominant tumour suppressor in GC and plays a crucial role in inflammatory response control in vivo. Manipulating the expression of BCL6B may provide a new entry point for GC treatment and regenerative medicine.

## Additional files

Additional file 1: **Figure S1.** Representative images showing immunostaining of Pan-Cytokeratin expression (Scale bar, 50 μm) from stomachs harbouring BaP-induced tumours (27 week) from *Bcl6b*^−/−^mice and WT controls (PDF 617 kb)

Additional file 2: **Figure S2.** Representative images showing Pan-Cytokeratin immunostaining (Scale bar, 50 μm) of stomachs harbouring BaP-induced tumours (25 week) from *Bcl6b*^−/−^mice and WT controls with or without 5-Aza treatment (PDF 750 kb)

## Data Availability

All data generated or analysed during this study are included in this published article

## Abbreviations

5-Aza: 5-Aza-2′-deoxycytidinel
BaP: Benzo[a]pyrene
BCL6: B cell CLL/lymphoma 6
BCL6B: B cell CLL/lymphoma 6 member B
BGS: Bisulphite genomic sequencing
ELISA: Enzyme-linked immunosorbent assay
GC: Gastric cancer
H&E staining: Haematoxylin and eosin staining
IHC: Immunohistochemistry
IL-1β: Interleukin 1 beta
IL-6: Interleukin 6
IL-8: Interleukin 8
mRNA: messenger RNA
qPCR: quantitative PCR
TNF-α: Tumour necrosis factor alpha
WT: Wildtype
