# Supplementary material for: Inhibition of Bcl6b promotes gastric cancer by amplifying inflammation in mice
Source: Cell Commun Signal. 2019 Jul 9;17:72. doi: 10.1186/s12964-019-0387-6 (PMC6617686; doi:10.1186/s12964-019-0387-6)
Supplement: Supplementary file 1 — Figure S1. Representative images showing immunostaining of Pan-Cytokeratin expression (Scale bar, 50 μm) from stomachs harbouring BaP-induced tumours (27 week) from Bcl6b−/−mice and WT controls (PDF 617 kb) [file 12964_2019_387_MOESM1_ESM.pdf]

Figure S1

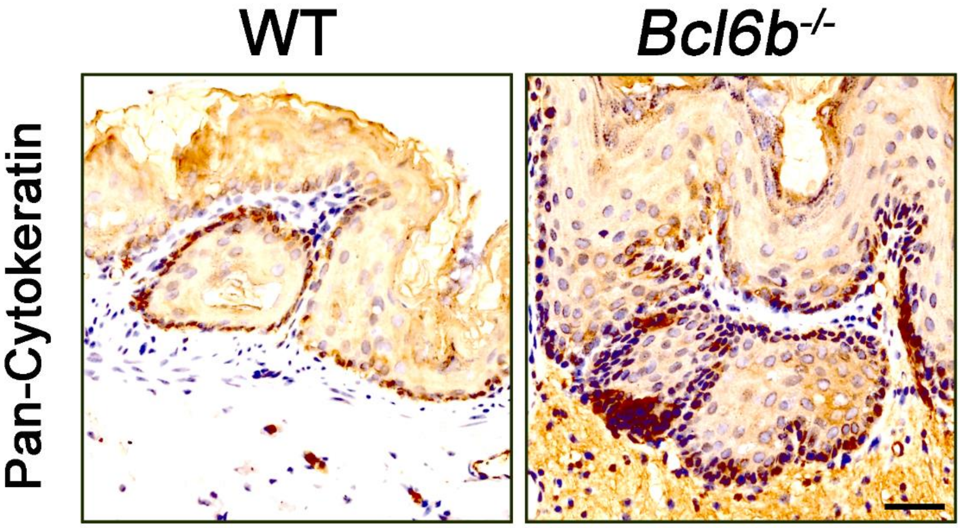

Figure S1. Representative images showing immunostaining of Pan-Cytokeratin expression (Scale bar, 50 μm) from stomachs harbouring BaP-induced tumours (27 week) from *Bcl6b*$^{-/-}$ mice and WT controls.
