# Supplementary material for: Inhibition of Bcl6b promotes gastric cancer by amplifying inflammation in mice
Source: Cell Commun Signal. 2019 Jul 9;17:72. doi: 10.1186/s12964-019-0387-6 (PMC6617686; doi:10.1186/s12964-019-0387-6)
Supplement: Supplementary file 2 — Figure S2. Representative images showing Pan-Cytokeratin immunostaining (Scale bar, 50 μm) of stomachs harbouring BaP-induced tumours (25 week) from Bcl6b−/−mice and WT controls with or without 5-Aza treatment (PDF 750 kb) [file 12964_2019_387_MOESM2_ESM.pdf]

Figure S2

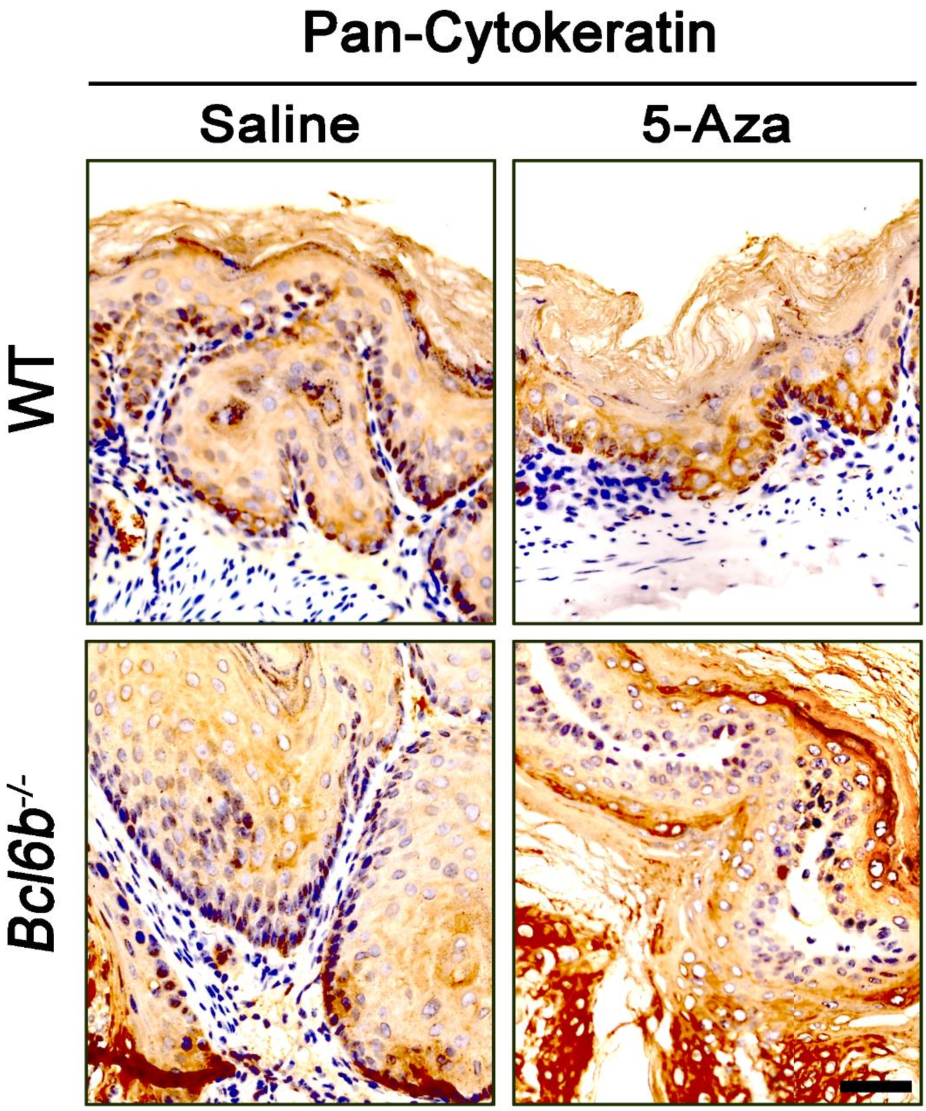

Figure S2. Representative images showing Pan-Cytokeratin immunostaining (Scale bar, 50 μm) of stomachs harbouring BaP-induced tumours (25 week) from *Bcl6b*$^{-/-}$ mice and WT controls with or without 5-Aza treatment.
